# Dealing with vaccine hesitancy in Africa: the prospective COVID-19 vaccine context

**DOI:** 10.11604/pamj.2021.38.3.27401

**Published:** 2021-01-05

**Authors:** Aanuoluwapo Adeyimika Afolabi, Olayinka Stephen Ilesanmi

**Affiliations:** 1 Department of Community Medicine, College of Medicine, University of Ibadan, Oyo State, Nigeria,; 2 Department of Community Medicine, University College Hospital, Ibadan, Oyo State, Nigeria

**Keywords:** COVID-19, vaccine hesitancy, Africa

## Abstract

The outbreak of the novel coronavirus disease (COVID-19) has resulted in many cases of morbidity and mortality across the globe, and the lack of the COVID-19 vaccine has contributed greatly to this experience. COVID-19 vaccines have currently been rolled out, and are available in some countries. However, strategies need to be put in place to prevent COVID-19 vaccine hesitancy (VH) especially in Africa; a continent where VH has been previously reported following the introduction of new vaccines. For this cause, we, therefore, recommend optimal community involvement in the structure and modalities for the delivery of the prospective COVID-19 vaccine. Also, feedback mechanisms for the acknowledgement of community efforts in previous health interventions should be improved upon to encourage the acceptance of the prospective COVID-19 vaccine. In addition, improved multi-sectoral collaboration should be initiated and promoted to enhance the acceptance of COVID-19 vaccines through the provision of more resources required to address COVID-19 VH. Furthermore, integration of the COVID-19 vaccine into the routine immunization schedule would strengthen the health system, improve uptake of the COVID-19 vaccine, and improve the health of all persons living on the African continent.

## Essay

The novel Coronavirus disease (COVID-19) pandemic is a global threat with which the entire globe is faced [1]. Since its outbreak in Wuhan city, China in December, 2019, 71,554,018 cases and 1,613,671 deaths have been recorded across 213 countries and five regions of the globe as of 23^rd^ December, 2020 [2]. On the African continent, 2,831,003 COVID-19 cases and 56,342 deaths have been recorded, with South Africa, Morocco, Egypt, Ethiopia, and Tunisia taking the lead [2]. The increasing number of fatalities have been due to the non-availability of any COVID-19 vaccine. At the early period of the COVID-19 pandemic when there was no known COVID-19 vaccine or treatment, herd immunity was suggested as a possible remedy for tackling SARS-CoV-2, the COVID-19 virus [3,4]. It was however estimated that herd immunity cannot be reached until 66.7% of the total population, vulnerable or healthy, gets exposed to SARS-CoV-2 [3]. Estimates from a study conducted across the West African sub-region revealed that for herd immunity to be achieved, 261 billion cases and nearly 5 million deaths would be recorded (at a case fatality rate of 2%) [4]. Thus, the proportion of cases and deaths globally cannot be kept within bearable limits if herd immunity is pursued. To arrest the increasing morbidity and mortality due to COVID-19, researches have been conducted for the development of a COVID-19 vaccine, and COVID-19 vaccines are currently available in some countries [5].

COVAX, a pillar of the Access to COVID-19 Tools (ACT) was launched by the World Health Organization, the European Commission, and France as a global response strategy to the COVID-19 pandemic [6]. COVAX was established as a global initiative to ensure both equitable and swift access to the COVID-19 vaccine in 190 countries across the globe, irrespective of their developmental phase or level of income [7]. Arrangements are underway to ensure the rolling out of the COVID-19 vaccine in the first quarter of 2021. For low-income countries who are not self-sufficient to purchase the COVID-19 vaccine, COVAX provides a lifeline and the only viable strategy to ensure a timely availability of the COVID-19 vaccine for their populace as developed countries [7]. Also, COVAX will bridge the inequality gap between the marginalized and other population groups in many African countries by ensuring that the COVID-19 vaccine is available to all persons [6,7]. In spite of these potential benefits presented by the COVID-19 vaccine, anecdotal evidence has reported the reluctance of many to accept the proposed COVID-19 vaccine, thus limiting the effectiveness of the COVID-19 outbreak response.

The COVID-19 outbreak response activities commenced in February, prior to the index case of COVID-19 in Africa. The response activities thus far have included, testing, border closure, school closure, recommendations on physical distancing, use of face masks, hand hygiene in public places, and public health campaigns on the existence of COVID-19. However, till date, many persons deny the existence of COVID-19, while others perceive it as a strategy for political corruption despite the public health campaigns [8]. Despite the misnomer, the existence of COVID-19 cannot be completely denied even among doubting individuals due to the large number of deaths that have been linked to COVID-19 in recent times. The denial of COVID-19 among many has influenced COVID-19 vaccine hesitancy (VH) following the knowledge of the availability of the COVID-19 vaccine in some countries. Aside the belief that the COVID-19 vaccine is political in nature, the lack of trust in the pharmaceutical industry, or its non-mandatory administration, the COVID-19 vaccine has been identified as the “mark of the beast” among religious folks [9]. These misconceptions could therefore hinder the promising successes which could be achieved through the prospective COVID-19 vaccine and the entire COVID-19 outbreak response. To address the factors which could hinder the effectiveness of the outbreak response, strategies need to be adopted to address COVID-19 VH.

**Vaccination programs and disease prevention:** vaccines have long been considered as one of the greatest achievements in attaining community and global health [10,11]. Due to the highly effective nature of vaccination programs on the African continent, a reduction in mortality and morbidity due to vaccine-preventable diseases as a result of high and sustainable uptake has been recorded especially among children [11,12]. Due to this result, lower costs are being incurred by households in the treatment of diseases [12]. Based on these benefits that are obtained with vaccination programs, an effective malarial vaccine has been suggested for the reduction of the malarial burden in malarial-endemic zones in Africa [13]. In lieu of this, the RTS, vaccine is being developed for efficacy against malaria. Similarly, experimental vaccines on the Ebolavirus disease are ongoing in five districts in Sierra Leone where most Ebolavirus cases have been reported. History of health interventions however posit that the introduction of new vaccines could be met by a number of challenges, one of which is VH [14].

**Vaccine hesitancy:** according to the World Health Organization, VH is defined as the delay in the acceptance or blunt refusal of vaccines, and has been identified as a growing trend in global health [15]. In the United States, Canada, California, and Europe, vaccine reluctance led to an increased vulnerability of unimmunized children in large, urban centers to the measles outbreak [16-18]. Many experiences and rumors have challenged the success and effectiveness of vaccination programs in Africa. The polio vaccine boycott in 2003-2004, prompted by distrust and fallacies resulted to a five-fold increase in the polio incidence in Nigeria between 2002 and 2006, and increased polio outbreaks in three non-African continents [19,20]. Due to the wrong perception of religious leaders, a polio vaccination program was rejected in Northern Nigeria [21]. Similarly, a mass deworming program was rejected in Ghana due to misconception among community members [22]. The rejection of vaccination programs in these instances thereby exposed more individuals to infectious illnesses and led to disease progression among ill individuals. These arrays of evidence thereby suggest that VH pose threat to individual, personal, and global health, as it delays the attainment of herd immunity for specific illnesses [20]. Myriads of experiences with infectious diseases and the weak health system on the African continent makes VH worth investigating, especially in the COVID-19 context.

**Possible causes of COVID-19 vaccine hesitancy in Africa:** COVID-19 VH could be considered as a cause-effect model, and its causes could be examined through multiple perspectives. Public distrust in the COVID-19 outbreak response on the African continent has been attributed to delayed response activities by the government of many African countries and public health experts [23]. Anecdotal reports obtained in Nigeria prior to the index case of COVID-19 in Africa on the 14^th^ February, 2020 revealed a laxity in the implementation of border closure. During this period, political moguls across Africa ensured the exit of their relatives from COVID-19 high-risk countries such as China, Germany, and the United States to Africa [23]. Such acts were not appreciated by individuals on the African continent because such prejudice led to the importation of COVID-19 to Africa and ultimately compromised the health of the masses [23]. In addition, community involvement was lacking while social distancing, hand hygiene, and other COVID-19 control measures were being implemented [23]. Furthermore, little was done by the African government to debunk theories on social and traditional media that the African continent was “immune” to COVID-19 due to the climatic conditions present therein [5,23]. Due to these reasons, many Africans displayed a lack of confidence in the government following reports of COVID-19 in Africa.

**Strategies for preventing the occurrence of COVID-19 vaccine hesitancy in Africa:** community participation presents a promising approach to addressing COVID-19 VH. Through the involvement of community stakeholders such as traditional heads, chiefs, opinion group leaders, and religious leaders, community members can be mobilized could be achieved [24]. Community mobilization in this regard is aimed at achieving two goals; firstly, to discredit false reports on the COVID-19 vaccine and ensure health education on the benefits of the COVID-19 vaccine. In this regard, the role of community health workers, community pharmacists, patent medicine vendors, and civil-based organizations come into play [25,26]. Secondly, community mobilization would contribute to an increased uptake of the prospective COVID-19 vaccine when available. Since community participation promotes a sense of ownership of any health intervention, if a traditional top-down approach is implemented while planning the COVID-19 vaccination activities in Africa, rejection of the vaccine would result in many settings, the purpose of procuring the vaccine would be defeated, and wastage of resources would ensue. Community participation would on the other hand enhance planning for the structure and modalities for making vaccines available in each African setting, and enable location of vaccine collection points in community-wide acceptable areas [24,25]. Also, community participation would forestall rivalry and strife which could occur secondary to siting vaccine collection points in other areas when community consent has not been obtained.

Feedback mechanisms have been shown to improve individual commitment towards any given project, and acknowledgement of previous contributions towards the success of any event is considered encouraging [27]. We need to celebrate the religious and community leaders who have participated in the success of past immunization programs like polio. Building on these truths to enhance community participation regarding the COVID-19 vaccine, expressing gratitude to communities for previous support obtained for the execution of health projects is mandatory. Community appreciation is not a herculean task which requires long-term planning. Rather, acknowledgement letters which have been duly signed could be forwarded to the community head, appreciation meetings where community members and leaders are present could be held in town halls, or incentives could be provided to community members who participated in the project. As the adage goes, “One good turn deserves another”, when community members become aware of how much value is placed on their participation, improved commitment is ascertained, and VH could be overcome while the COVID-19 vaccine is being hastened.

With promising results from three ongoing COVID-19 vaccine trials, multisectoral collaboration is a sure key to enhancing COVID-19 vaccine uptake and combating VH [27,28]. Due to the low-resourced nature of many countries on the African continent, the national government may not be able to solely bear the COVID-19 vaccine expenditure [28]. Although, subsidization of the cost of the COVID-19 vaccine has been put in place by COVAX for low-income countries, more support is needed to enable large-scale purchase of the COVID-19 vaccine by African countries. Therefore, collaboration of the both private and public sectors could enhance the affordability of the COVID-19 vaccine by the government in African countries. Multi-sectoral involvement in the COVID-19 outbreak response in Nigeria has yielded optimal results in the provision of more personal protective equipment, testing kits, logistics, and financial resources; however, collaboration to address the COVID-19 VH should be prioritized at this critical period [29]. All sectors in each country on the African continent should be responsive towards improving health education on the importance of the COVID-19 vaccine. Community informants could also be employed to notify sectoral representatives in each county or local government area to ensure that false pieces of information regarding the COVID-19 vaccine are addressed immediately [24]. In addition, multi-sectoral collaboration could be optimized to conduct research on COVID-19 VH or COVID-19 vaccine acceptance among social media users [30]. Knowledge gain in this regard would be helpful to implement strategies to correct COVID-19 VH misconceptions.

Integration of the prospective COVID-19 vaccine into the existing healthcare services presents a promising strategy to overcoming VH to improve vaccine uptake [30]. Due to the utilization of existing healthcare resources, the horizontal system approach prevents wastage of resources, both individual and healthcare [30]. It is known that certain costs are attached to registration at health facilities and could be doubled if the COVID-19 vaccination is implemented vertically; however, integration of the COVID-19 vaccine prevents the placement of double burden of care for each member of the household, as well as the waiting time for vaccine collection. In addition, integration of the COVID-19 vaccine permits its decentralization to promote proximity to residential areas in a bid to reduce the cost of transportation of many individuals [24]. The National Primary Health Care Development Agency should be both responsive and responsible in this regard. The routine immunization activity in Nigeria, for instance, has been faced with the challenge of VH, secondary to vaccine misconceptions, long distance, and unavailability of parents. These challenges could however be overcome when the COVID-19 vaccine is readily available and accessible to community members through an enhanced cold chain.

## Conclusion

The COVID-19 pandemic has resulted in many unplanned mortality and fatality cases. To prevent more cases in this regard, the COVID-19 vaccine has been developed and has been rolled out for use in some countries. Similar to previous health events, COVID-19 VH has been hypothesized on the African continent despite the benefits it presents. To overcome VH, we recommend optimal community involvement in the structure and modalities for vaccine delivery. Also, feedback mechanisms for the acknowledgement of community efforts in previous health interventions should be improved to encourage their acceptance of the COVID-19 vaccine. In addition, improved multi-sectoral collaboration would enhance acceptance of COVID-19 vaccines through the provision of more resources required to address COVID-19 VH. Furthermore, integration of the prospective COVID-19 vaccine into the routine immunization schedule would strengthen the health system, improve uptake of the COVID-19 vaccine, and improve the health of all persons living on the African continent. Further research on COVID-19 VH should be considered by researchers during this notable period.
